# miR-24-3p Regulates Epithelial–Mesenchymal Transition and the Malignant Phenotype of Pancreatic Adenocarcinoma by Regulating ASF1B Expression

**DOI:** 10.1007/s10528-022-10278-5

**Published:** 2022-09-17

**Authors:** Wentao Huang, Tiansheng Lin, Long Huang, Junyi Wu, Jiaming Hong, Funan Qiu, Yifeng Tian, Yaodong Wang

**Affiliations:** Department of Hepatobiliary and Pancreatic Surgery, Fujian Provincial Hospital, Shengli Clinical Medical College of Fujian Medical University, Fujian Medical University, NO.134, Dongjie Street, Gulou District, Fuzhou, 350001 Fujian China

**Keywords:** Pancreatic ductal adenocarcinoma, Epithelial–mesenchymal transition ASF1B, miR-24-3p

## Abstract

Anti-silencing function protein 1 homolog B (ASF1B) has been implicated in the occurrence and development of cancers. The present work explored the functional role and the expression regulation of ASF1B in pancreatic ductal adenocarcinoma (PDAC). Based on the real-time quantitative PCR (qRT-PCR) and immunohistochemistry (IHC), ASF1B was significantly upregulated in PDAC tissues. High expression of ASF1B was associated with a poor overall survival (OS) and recurrence-free survival (DFS) in the PDAC patients. ASF1B also showed a relatively higher expression in PDAC cells (AsPC-1, PANC-1) when compared with human pancreatic ductal epithelial cells (HPDFe-6). CCK8 and clone formation assay demonstrated that silencing ASF1B impaired the proliferation in PANC-1 and AsPC-1 cells, and Annexin V-PI staining showed an increased level of apoptosis upon ASF1B silencing. ASF1B silencing also suppressed the migration and invasion in PDAC cells, as revealed by Transwell assays. We further showed that miR-24-3p was downregulated in PDAC tissues and cells, which functionally interacted with ASF1B by dual-luciferase reporter assay. miR-24-3p negatively regulated ASF1B expression to modulate the malignant phenotype of PDAC cells. ASF1B shows high expression in PDAC, which promotes the malignancy and EMT process of PDAC cells. miR-24-3p is a negative regulator of ASF1B and is downregulated in PDAC cells. Our data suggest that targeting ASF1B/miR-24-3p axis may serve as an intervention strategy for the management of PDAC.

## Introduction

Pancreatic ductal adenocarcinoma (PDAC) is one of the most lethal malignant tumors with poor prognosis. Despite substantial improvements in survival rates in other cancers, PDAC remains as a serious challenge with the lowest survival rate since the 1960s (Ansari et al. 2016). Due to the lack of early diagnostic marker, PDAC is associated with the high mortality especially for the patients diagnosed in advanced stages, and its 5-year survival is as low as 10% (Bray et al. 2018). The molecular mechanisms underlying its malignant progression or metastasis remain unclear, and a comprehensive knowledge of the pathogenic progression of PDAC is of great significance for its diagnosis and the formulation of novel therapeutic strategies.

EMT (epithelial–mesenchymal transition) represents a critical biological process in the embryogenesis and organogenesis (Lamouille et al. 2014). In tumor biology, EMT has been recognized as a key event contributing to the invasion and metastasis of cancer cells (Chaudhry et al. 2014; Mittal 2018). This process is accompanied by the downregulation of diverse epithelial markers and the upregulation of mesenchymal markers, such as the decrease of E-cadherin level and an increase mesenchymal marker levels (N-cadherin or fibronectin) (Hugo et al. 2007). Owing to the extremely high malignancy of pancreatic cancer, metastasis and drug resistance can progress rapidly, and the EMT process is closely related to its metastasis and drug resistance development (Zhou et al. 2017; Sato et al. 2020).

Anti-silencing function 1 (ASF1) is a molecular chaperone of histone H3-H4 involved in DNA repair, replication, as well as transcriptional modulation (Paul et al. 2016). ASF1 plays an essential role in regulating the nucleosome organization through promoting histone deposition and histone exchange (Natsume et al. 2007). Abnormal ASF1 upregulation may facilitate cancer development and progression (Hu et al. 2021). ASF1 consists of two isoforms, ASF1A and ASF1B (Abascal et al. 2013), which share 70% homology in sequence but differ functionally. It has been shown that ASF1A is involved in the regulation of interphase (except DNA synthesis phase) and DNA damage repair process, whereas ASF1B is engaged in mitosis and cell division (Groth et al. 2005). Previous studies suggest that abnormal ASF1B expression contributes to the occurrence and progression of different cancers, including cervical cancer (CC), thyroid cancer (TC), breast cancer (BC), prostatic cancer (PCa) and lung cancer (LC) (Liu et al. 2020; Corpet et al. 2011; Ma et al. 2021; Zhang et al. 2021; Han et al. 2018). In lung cancer, ASF1B was reported to regulate EMT and metastasis (Wu and Jie 2021). However, its biological function and expression regulation in PDAC have not been reported.

MicroRNAs (miRNAs) are an important class of noncoding RNAs (ncRNAs), which modulate the translation or stability of target mRNAs through binding to a certain region in the 3′ noncoding region (3′ UTR) (Cannell et al. 2008). Aberrant miRNA expression has been reported as contributing factor in cancer progression by promoting the expression of pro-oncogenes or suppressing the expression of tumor-suppressor genes (Zan et al. 2019). Several miRNAs have been found to be engaged in the EMT process through regulating important transcription factor or EMT-related genes (Saberinia et al. 2020; Zmarzły et al. 2021). For example, An et al. demonstrated that miR-203a-3p can target and regulate SLUG mRNA; thereby, inhibiting the cell proliferation and inducing apoptosis in pancreatic cancer cells (An and Zheng 2020). Peng et al. reported that miR-148a inhibits the EMT and the invasion ability of pancreatic ductal adenocarcinoma cells by regulating WNT10B (Peng et al. 2017). miR-24-3p has been shown to inhibit the progression of pancreatic ductal adenocarcinoma by downregulating LAMB3 (Huang et al. 2019). miR-24-3p also regulates the EMT process in lung cancer and retinoblastoma (Jing et al. 2020; Luan et al. 2021), but its role in PDAC remains to be elucidated.

In this study, ASF1B was identified as a target of miR-24-3p in PDAC cells. ASF1B showed upregulation while miR-24-3p showed downregulation in PDAC tissues. We further demonstrated that miR-24-3p regulates the expression of ASF1B, thereby modulating the malignancy of PDAC cells. These data lay theoretical foundation for the intervention of miR-24-3p/ASF1B axis in PDAC as a potential intervention strategy.

## Materials and Methods

### Online Bioinformatics Analysis

ASF1B expression levels in PDAC samples and normal samples were retrieved from TCGA database and GETx project using GEPIA online resources (http://gepia.cancer-pku.cn/). A total number of 179 tumors along with 171 normal tissues were included in the analysis. The association of ASF1B expression level with the overall survival of PDAC patients was analyzed using Kaplan–Meier Plotter in GEPIA online resources, with a total of 178 cases being included (Ye et al. 2022). Four databases including Starbase (https://starbase.sysu.edu.cn/starbase2/index.php), Targetscan (http://www.targetscan.org/vert_71/), miRDB (http://mirdb.org/) and miRWalk (http://mirwalk.umm.uni-heidelberg.de/) were employed to predict the miRNAs with possible binding sites with ASF1B mRNA.

### Collection of Tissue Samples

A total number of 120 PDAC tissues and the matched para-neoplastic tissues were collected by surgical resection at the Department of Hepatobiliary Pancreatic Surgery, Fujian Provincial Hospital. All the patients were primarily diagnosed with PDAC through postoperative pathology, without preoperative chemotherapy or radiotherapy. All the collected tissues were snap-frozen in liquid nitrogen and stored at − 80 °C deep freezer (Liu et al. 2021). All the patients signed the informed consent. This study gained approval from the research ethics committee of Fujian Provincial Hospital.

### Immunohistochemistry (IHC)

After paraffin embedding, the tissues were cut into 5 μm sections and processed through xylene deparaffinage and gradient alcohol dehydration. Antigen retrieval was perfromed by high pressure cooking and tryptic digestion, and 6% normal goat serum was used for blocking (Invitrogen, CA, USA) for half an hour at room temperature. The tissue sections were incubated with rabbit anti-human ASF1B polyclonal antibody (1:500, Abcam, Cambridge, UK) at 4 °C overnight. After three washes with PBS, the sections were further incubated with biotin-labeled goat antirabbit IgG secondary antibody (1:1000, Abcam, Cambridge, UK) at room temperature for 1 h. Goat IgG was used as negative control. After three washes with PBS, each section was stained by DAB for 5 min, and the slides were differentiated in 2% hydrochloric acid alcohol, bathed in PBS and counterstained with hematoxylin (Cwbiochem, Beijing, China). Positive staining was defined as brown or yellow particles within nuclei or cytoplasm, and was quantified by the immunoreactive score (IRS) method: staining intensity (SI) score 0, 1, 2, and 3 points stand for no signal, light yellow, brownish yellow, brownish brown staining, separately. The positively-stained cell percentage (PP) scores were rated as: 0-negative, 1-PP 1–10%, 2-PP 11–50%, 3-PP 51–75%, and 4-PP 76–75%. IRS = SI score × PP score, IRS ≥ 3 is considered as positive, IRS < 3 is considered to be negative. The staining scores were semi-quantitatively validated by 2 senior pathologists using a double-blind method (Wu et al. 2020).

### Cell Culture

All the cell lines were obtained from Shanghai Cell Bank (http://www.bluefcell.com/?bd_vid=7599772084905760421). Human pancreatic cancer cells PANC-1 and AsPC-1, and human pancreatic ductal epithelial cells HPDE-6 were cultured in DMEM containing 10% fetal bovine serum (FBS; Gibco), 100 U/ml of penicillin and 100 μg/ml of streptomycin in a humidified incubator containing 5% CO2 at 37 °C (Liu et al. 2021).

### Small RNA Interference (siRNA)

The siRNAs targeting ASF1B was synthesized by Guangzhou Ribo Company. Sequences for si-ASF1B 1#: 5′-GGUUCGAGAUCAGCUUCGAGU-3′; 2#: 5′-GCAGGUACUUCAGUUCUAAAU-3′; 3#,5′-GUUAGUAGGUAGACUUA GAU-3′. si-NC sequence: 5′-TCTCCGAACGTGTCACGT-3′. Cells at logarithmically growing phases were transfected with 100 nM ASF1B siRNA or si-NC. Using Lipofectamine 3000 (Invitrogen, USA) following the manufacture’s instruction. qRT-PCR and WB assays were performed to examine the knockdown efficiency 48 h after transfection (Ran et al. 2018).

### Cell Transfection

AsPC-1 and PANC-1 cells were seeded at a density of 2 × 10^5^/well into 6-well plates and cultured until reaching approximately 70% confluence. Transfection was performed using Lipofectamine 3000 reagent (Invitrogen, USA). miR-24-3p mimic or its inhibitor was transfected into AsPC-1 cells for overexpression or knockdown, respectively. 6 h after transfection, the medium was replaced with fresh DMEM with 10% FBS. Cells were subjected to further experiments 48 h after transfection (He et al. 2019).

### Cell Counting Kit-8 (CCK8) Assay

AsPC-1 and PANC-1 cells with different treatments were seeded at a density of 2 × 10^3^/well in 96-well plates. CCK-8 reagent (10 μl, Beyotime, Shanghai, China) was added to each well at indicated time point and the cell culture was incubated for 2 h under 37 °C with 5% CO_2_. The absorbance (OD) was recorded with a microplate reader (Bio-Rad, USA) at 450 nm (Chen and Zhou 2018).

### Colony Formation Assay

AsPC-1 and PANC-1 cells were seeded at a density of 2 × 10^3^/well into 6-well cell plates, followed by 2-week incubation under 37 °C and 5% CO2 conditions. After the single colonies became visible, the cells were fixed with 4% paraformaldehyde (PFA) for a 15 min. After the removal of the fixative, Giemsa stain (Sigma, Germany) was added for 20 min incubation. After two washes with PBS, the samples were air dried and observed under a microscope. Colonies with more than 10 cells were included in colony counting at×200 magnification, and the colony formation ratio was determined as (clone number/inoculated cell number)×100% (Fang et al. 2020).

### Annexin V-PI Staining for Apoptosis Detection

The 0.25% trypsin was utilized to detach logarithmically growing AsPC-1 and PANC-1 cells. Annexin V Apoptosis Detection Kit (BD Biosciences, PharMingen, San Jose, CA, USA) was used to detect apoptosis according to the manufacturer's instructions. In brief, 5 μL Annexin V-FITC and 5 μL PI reagent were added to 1000 μL cell suspension with 1 million cells, and the cell mixture was incubated for 30 min in the dark. Stained cells were centrifuged and washed twice with PBS and re-suspended in 400 μL PBS. The percentage of apoptotic events was analyzed by BD FACS CantoTM II Flow Cytometer (BD Biosciences) (Li et al. 2020).

### Transwell Migration and Invasion Assay

Cells at logarithmic growing phase were starved for 24 h, followed by trypsin digestion and re-suspended at a density of 2 × l0^5^/ml. The invasion and migration assays were performed with using the Transwell chambers (pore size, 8 μm; Coring, NY, USA). For the invasion assay, the upper chamber was coated with Matrigel (Corning, CA, USA). 1 × 10^5^ cells were inoculated into the Transwell upper chamber in 200 µl serum-free medium, whereas the bottom chamber was filled with 600 µl medium containing 30% fetal bovine serum. After 18 h, the cells on the upper chamber membrane were fixed with 4% PFA, followed by 20 min staining of 0.5% crystal violet (Sigma, Germany). After three washes with PBS, the cells were photographed under an inverted microscope at×200 magnification. For each sample, the number of cells in 5 random fields were counted (Marshall 2011).

### Dual Luciferase Assay

The Bioinformatics tools miRDB/miRWalk predicted the presence of miR-24-3p’s binding sites in ASF1B mRNA 3′UTR. The luciferase reporter containing wild-type (WT) or mutated (MUT) ASF1B fragment (ASF1B-WT or ASF1B-MUT), miR-24-3p mimic and miRNA control (miR-NC) were purchased from Genescript (Piscataway, NJ). AsPC-1 and PANC-1 cells were co-transfected with luciferase reporter containing either ASF1B–MUT or ASF1B-WT, and miR-24-3p mimic or miR-NC by Lipofectamine 3000 reagent (Invitrogen, USA). 48 h after transfection, the luciferase activities were measured using Dual-Luciferase Reporter Assay System (Promega, Madison, WI, USA). The relative firefly luciferase activity in the reporter plasmid was normalized to that of Renilla luciferase (hRlucneo) control plasmid (Liu et al. 2021).

### Quantitative Real-Time Fluorescence PCR (qRT-PCR)

Trizol reagent (Invitrogen, Carlsbad, CA, USA) was utilized to extract total RNA from tissues and cells, and the quality of purified RNA samples was assessed using a micro-spectrophotometer (NanoDrop 200C, Wilmington, USA) at 260–280 nm. Using the High Capacity cDNA Reverse Transcription kit (Applied Biosystems, CA, USA), cDNA was prepared from 2 μg of total RNA. For amplification, the qPCR reaction comprised 1 μl of total cDNA template, forward and reverse primers (10 nmol/L, 1 μl each), 10 μl of 2×SYBR Premix EXTaq (Takara, Tokyo, Japan) and 7 μl of DEPC water in a total volume of 20 μl. The following PCR conditions were used: 3-min pre-denaturation at 95 °C, 40 cycles of 15-s denaturation at 95 °C; 35-s annealing at 60 °C and 30-s extension at 72 °C. GAPDH and U6 were used as the internal references, and 2-∆∆Ct approach was employed for normalization (Schmittgen and Livak 2008). The primer sequences were listed below: ASF1B: F 5′-TCCGGTTCGAGATCAGCTTC-3′, R 5′- GTCGGCCTGAAAGACAAACA-3′; U6: F 5′-GCTTCGGCAGCACATATA CTAA-3′ R 5′-CGAATTTGCGTGTCATCCTT-3′; GAPDH: F 5′-GGAGCG AGATCCCTCCAAAAT-3′, R 5′-GGCTGTTGTCATACTTCTCATGG-3′; miR-24-3p: F 5′-AGTGGCTCAGTTCAGCA-3′, R 5′-CCAGTTTTTTTTTTTTTT TCTGTTCCT-3′.

### Western-Blotting (WB)

RIPA lysis buffer (Beyotime, Shanghai, CHN) was used for cell lysis on ice for 15 min. The protein quantification was qualified using a BCA assay kit (Beyotime, Shanghai, China). 10 μg of protein sample was separated in 12% SDS-PAGE, and then transferred to polyvinylidene difluoride (PVDF) membrane (Merck Millipore, MA, USA). 5% defatted milk was utilized to block membrane for 1 h at ambient temperature. The membrane was incubated with primary antibodies (Abcam) ASF1B (1:800), vimentin (1:1,000), N-cadherin (1:500), and E-cadherin (1:1,000) overnight at 4 °C. After washes, the membrane was further incubated with secondary antibodies (abcam, 1:2,000) for 1 h at ambient temperature. Protein bands were visualized using an enhanced chemiluminescence kit (Santa Cruz, TX, USA) and photographed on a gel imager system (Bio-Rad, Hercules, CA, United States). The densitometry analysis was performed with Image J v1. 52 software (Bethesda, MD, USA) (Hamano et al. 2011).

### Statistical Analysis

GraphPad Prism 8.00 statistical software was used for statistical analysis, and the results were presented as mean ± SD. Statistical difference between two groups was analyzed using unpaired Student’s t tests, and the comparisons among multiple groups were analyzed using one-way analysis of variance (ANOVA) with Tukey’s post hoc test. The data at multiple time points were analyzed using two-way ANOVA. Kaplan–Meier curve and log-rank test were used to compare the cumulative survival rates in PDAC patients. *P* < 0.05 stood for statistical significance.

## Result

### ASF1B is Significantly Upregulated in PDAC Patients and is Associated with a Poor Prognosis

**Fig. 1:**
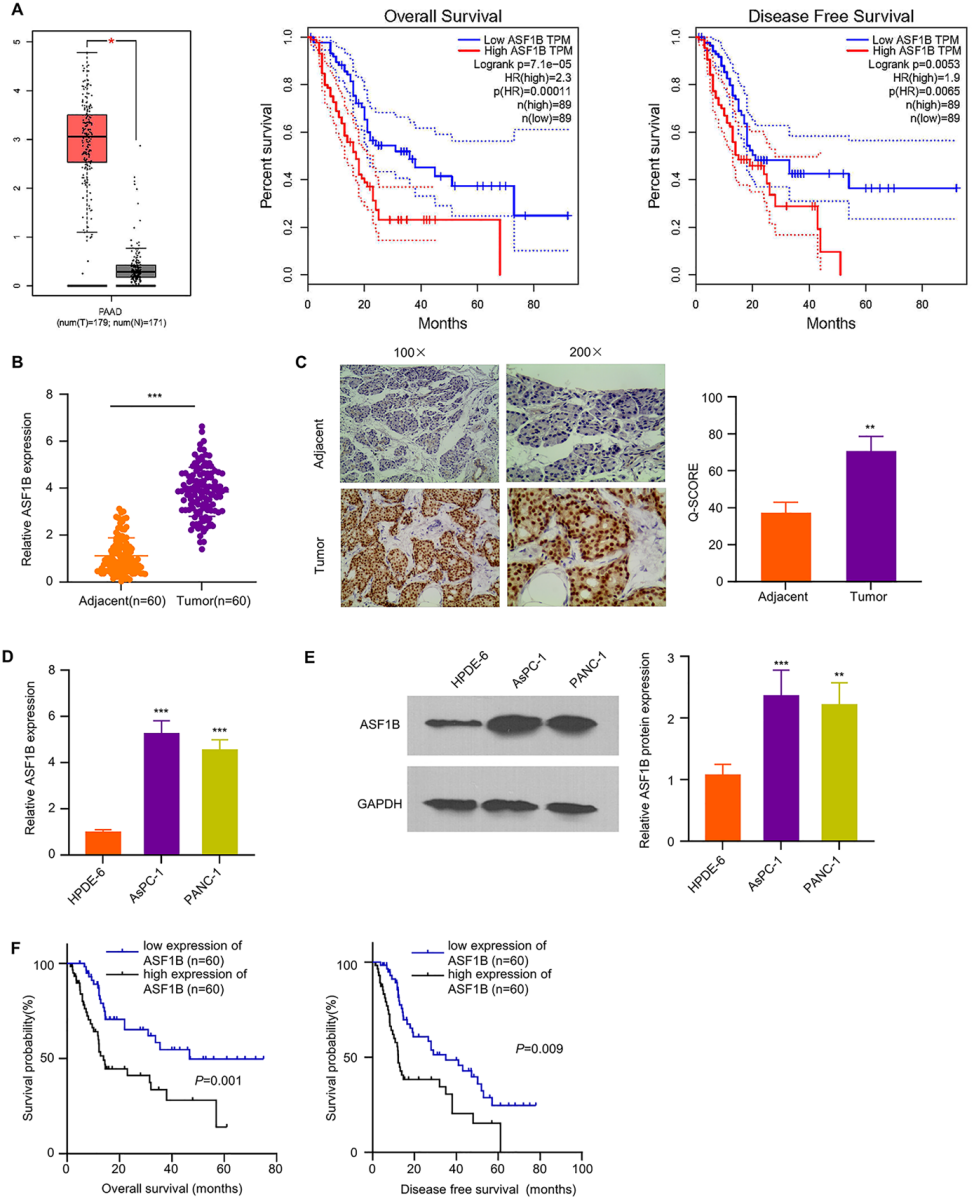
ASF1B expression is significantly upregulated in PDAC and its high expression is correlated with poor prognosis. **A** Bioinformatics analyses were performed to ASF1B expression level and the association of ASF1B level with PDAC survival using dataset of TCGA-PDAC from GEPIA. **B** ASF1B mRNA levels in 120 samples of PDAC tissues and para-neoplastic normal tissues were analyzed by qRT-PCR. **C** ASF1B protein level in PDAC tissues and para-neoplastic normal tissues was examined by immunohistochemistry. **D** ASF1B mRNA levels in PDAC cell lines (PANC-1 and AsPC-1) and human pancreatic ductal epithelial cells (HPDE-6) were detected by qRT-PCR. **E** ASF1B protein levels in PDAC cell lines (PANC-1 and AsPC-1) and human pancreatic ductal epithelial cells (HPDE-6) were detected by Western blot. **F** Association of ASF1B expression level and the survival (OS and DFS) in 120 PDAC patients was analyzed through Kaplan–Meier curve and log-rank test. The results are summarized as mean ± SD. *p < 0.05, **p < 0.01, ***p < 0.001

We first retrieved data of ASF1B expression in PDAC samples and normal pancreatic tissues from the TCGA-PDAC and GTEx dataset using GEPIA database (http://gepia.cancer-pku.cn/). The results showed that SF1B is significantly upregulated in PDAC patients and Kaplan–Meier curve analysis revealed that a high level of ASF1B expression was associated with a poorer prognosis in PDAC patients (Fig. 1A). We further compared the expression of ASF1B in 120 pairs of PDAC tissues and adjacent normal samples by IHC staining and qRT-PCR. These analyses confirmed the upregulation of ASF1B in PDAC tissues at protein and mRNA levels (Fig. 1 B and C). In human PDAC cell lines (AsPC-1 and PANC-1), ASF1B also showed a relatively higher level than that of pancreatic ductal epithelial cells HPDE-6. (Fig. 1D and E). In addition, the PDAC patients were divided into ASF1B high expression and low expression groups (*n* = 60 in each) based on the median level of ASF1B expression. A high ASF1B level was significantly associated with more advanced TNM stage, larger tumor size and more lymph node metastasis while such association was not observed in age and gender (Table 1). Further, Kaplan–Meier (KM) curves showed that high ASF1B expression was also correlated with a poorer overall survival and disease-free survival in PDAC patients (Fig. 1F). Overall, these data suggest that a high ASF1B expression level contributes to a poorer prognosis in PDAC patients.

**Table 1:** The associations of clinicopathological parameters of PDAC patients with ASF1B expression level

Chinicopathological characteristics	Total	ASF1B expresstion		P value
		high	low	
Gender				
Male	56	29	27	0.714
Female	64	31	33	
Age(years)				
≤ 60	65	31	34	0.583
> 60	55	29	26	
Tumor size				
T1	31	7	24	0.001**
T2	32	17	15	
T3	28	15	13	
T4	29	21	8	
Lymph node metastasis				
Positive	44	41	23	0.001**
Negtive	36	19	37	
TNM stages				
I	31	8	23	0.007**
II	28	14	14	
III	29	16	13	
IV	32	22	10	

*p < 0.05, **p < 0.01, ***p < 0.001

### ASF1B Silencing Inhibits Tumor Cell Growth, Invasion and Migration

**Fig. 2:**
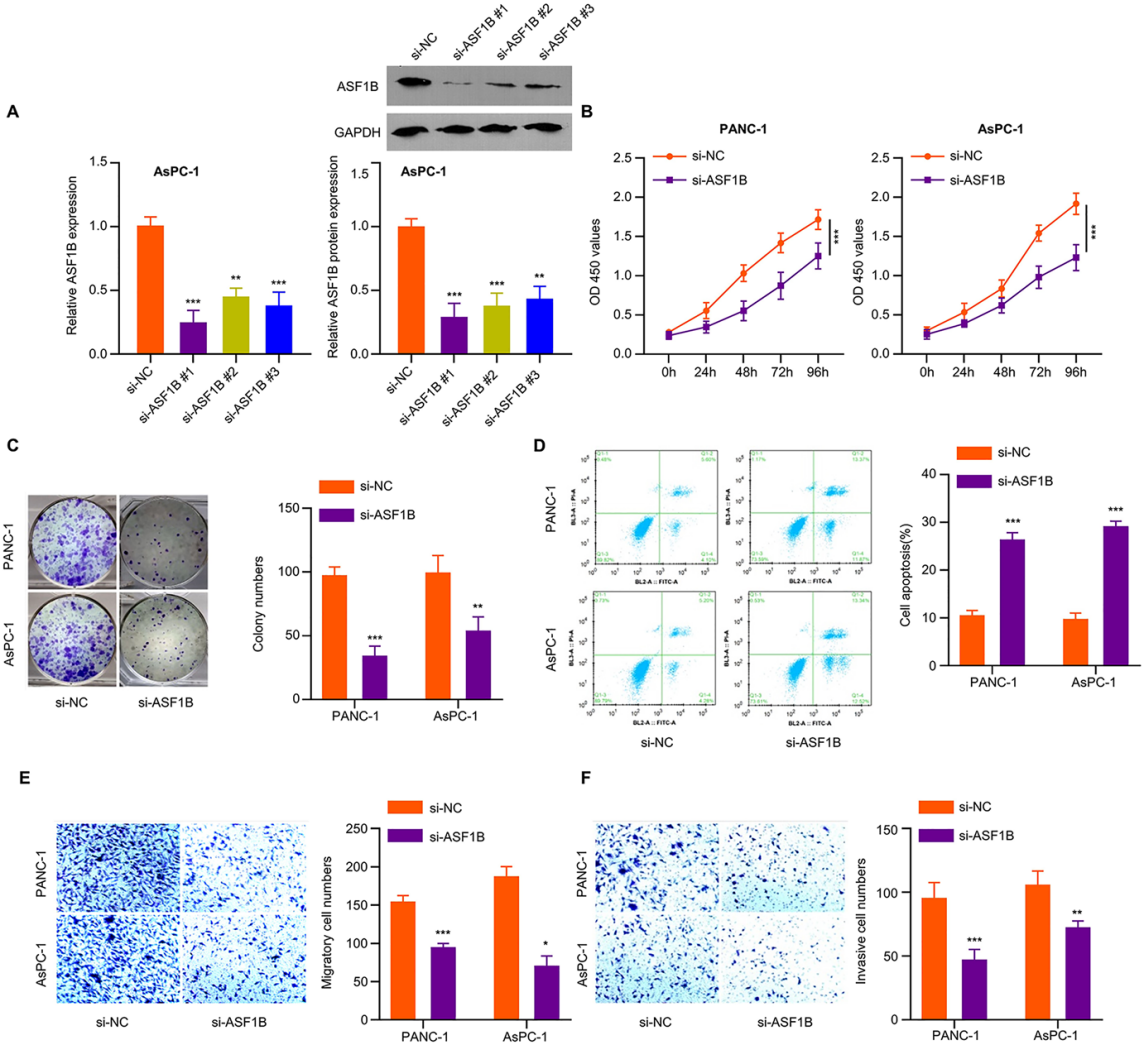
Silencing ASF1B inhibits the cell proliferation, migration and invasion of PDAC. **A** AsPC-1 cells were transfected with ASF1B-siRNA-1/2/3, and ASF1B mRNA and protein levels were examined by qRT-PCR and WB. **B** CCK-8 proliferation assay in PDAC cells upon ASF1B silencing. **C** Colony formation assay in PDAC cells upon ASF1B silencing. **D** Apoptotic events in PDAC cells upon ASF1B silencing were measured by Annexin V PI staining. **E** Transwell migration assay and **F** invasion assay were performed in PDAC cells after ASF1B silencing. The data are summarized as mean ± SD. *p < 0.05, **p < 0.01, ***p < 0.001

To investigate the biological role of ASF1B in PDAC, we applied siRNA to silence ASF1B in AsPC-1 cells. The knockdown efficiencies of ASF1B-siRNA-#1, #2, and #3 were examined by qRT-PCR and Western blot, which showed the strongest silencing effect using siRNA #1 (Fig. 2A). Therefore, ASF1B-siRNA-#1 was selected for the subsequent experiments. CCK-8 proliferation assay and colony forming assay both showed a significant impairment in cell proliferation upon ASF1B knockdown (Fig. 2B and 2C). Flow cytometric analysis revealed that silencing ASF1B induced apoptosis in PDAC cell lines (Fig. 2D), and Transwell migration and invasion assays demonstrated a suppression of cellular migration and invasion upon ASF1B silencing (Fig. 2E and F). Together, the above data imply that ASF1B is required to maintain the malignant phenotype of PDAC cells.

### miR-24-3p Negatively Regulates ASF1B and is Downregulated in PDAC Tissues and Cells

**Fig. 3:**
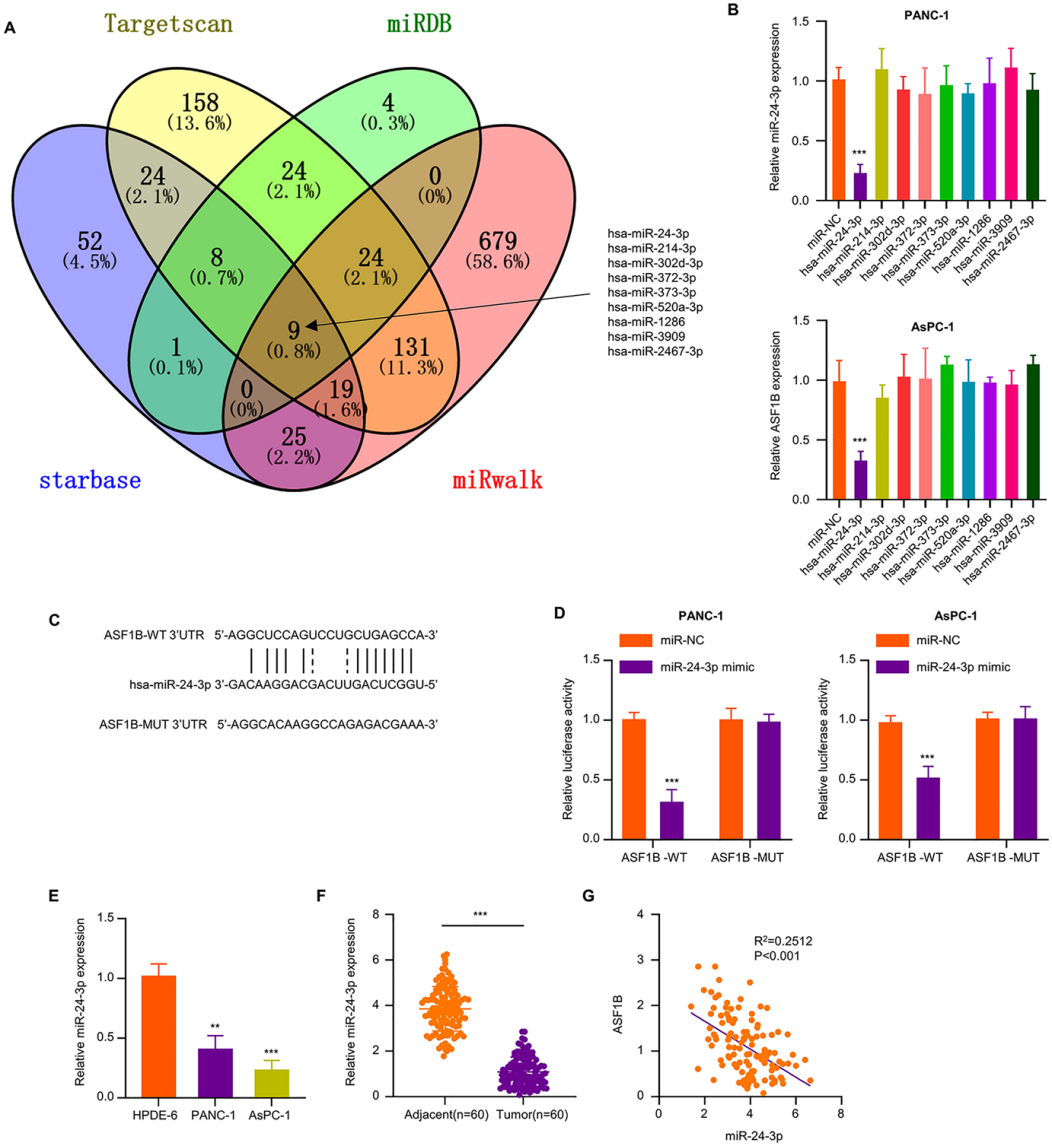
miR-24-3p negatively regulates ASF1B in PDAC cells. **A** The miRNA targets of ASF1B were predicted using online databases “starbase”, “Targetscan”, “miRDB”, and “miRwalk”. 9 miRNAs (hsa-miR-24-3p, hsa-miR-214-3p, hsa-miR-302d-3p, hsa-miR-372-3p, hsa-miR-373-3p, hsa-miR-520a-3p, hsa-miR-1286, hsa-miR-3909, hsa-miR-2467-3p) were predicted to target ASF1B mRNA. **B** qRT-PCR analysis of ASF1B level in AsPC-1 cells after the transfection of different miRNA mimics. **C** Schematics of binding sites for miR-24-3p in ASF1B mRNA 3'UTR. **D** Dual-luciferase reporter assay using vector containing WT binding sites in ASF1B 3'UTR or mutated (MUT) binding sites. **E** miR-24-3p expression levels in PC cells (AsPC-1 and PANC-1) and normal pancreatic epithelial cells (HPDE-6) were measured by qRT-PCR. **F** miR-24-3p expression in PDAC tissues and adjacent noncarcinoma samples was measured by qRT-PCR. **G** Spearman’s correlation coefficient analysis of the expression levels of ASF1B and miR-24-3p in PDAC tissues. The data are summarized as mean ± SD. *p < 0.05, **p < 0.01, ***p < 0.001

To explore the miRNAs regulating ASF1B expression, we searched four databases (Starbase/Targetscan/miRDB/miRWalk) which predicted that there were 9 potential miRNAs containing potential binding sites for the 3′UTR of ASF1B mRNA (Fig. 3A). Further, the mimics of the above 9 miRNAs and miR-NC were transfected into AsPC-1 cells, and qRT-PCR analyses showed that only miR-24-3p mimic was able to cause the reduction in ASF1B mRNA level (Fig. 3B). Next dual-luciferase reporter assay using reporter containing WT 3′UTR of ASF1B mRNA (ASF1B-WT) or the sequence with mutated miR-24-3p binding sites (ASF1B-MUT) were performed to validate the functional interaction between ASF1B mRNA and miR-24-3p. The overexpression of miR-24-3p inhibited the luciferase activity of WT reporter while the inhibitory effect was abrogated in the mutated reporter, indicating that the binding ability of miR-24-3p to ASF1B 3'UTR region is essential for their interaction (Fig. 3C and D). We also analyzed the expression level of miR-24-3p in PDAC tissues and cell lines and found that, miR-24-3p level was largely reduced in PDAC tissues and cell lines (Fig. 3E and F). Spearman's correlation coefficient analysis further demonstrated a negative correlation between ASF1B and miR-24-3p expression levels in PDAC tissues (Fig. 3G). Together, these data suggest that miR-24-3p negatively regulates ASF1B and is downregulated in PDAC tissues and cells.

### Overexpression of miR-24-3p Suppresses ASF1B Level in PC Cell Lines

**Fig. 4:**
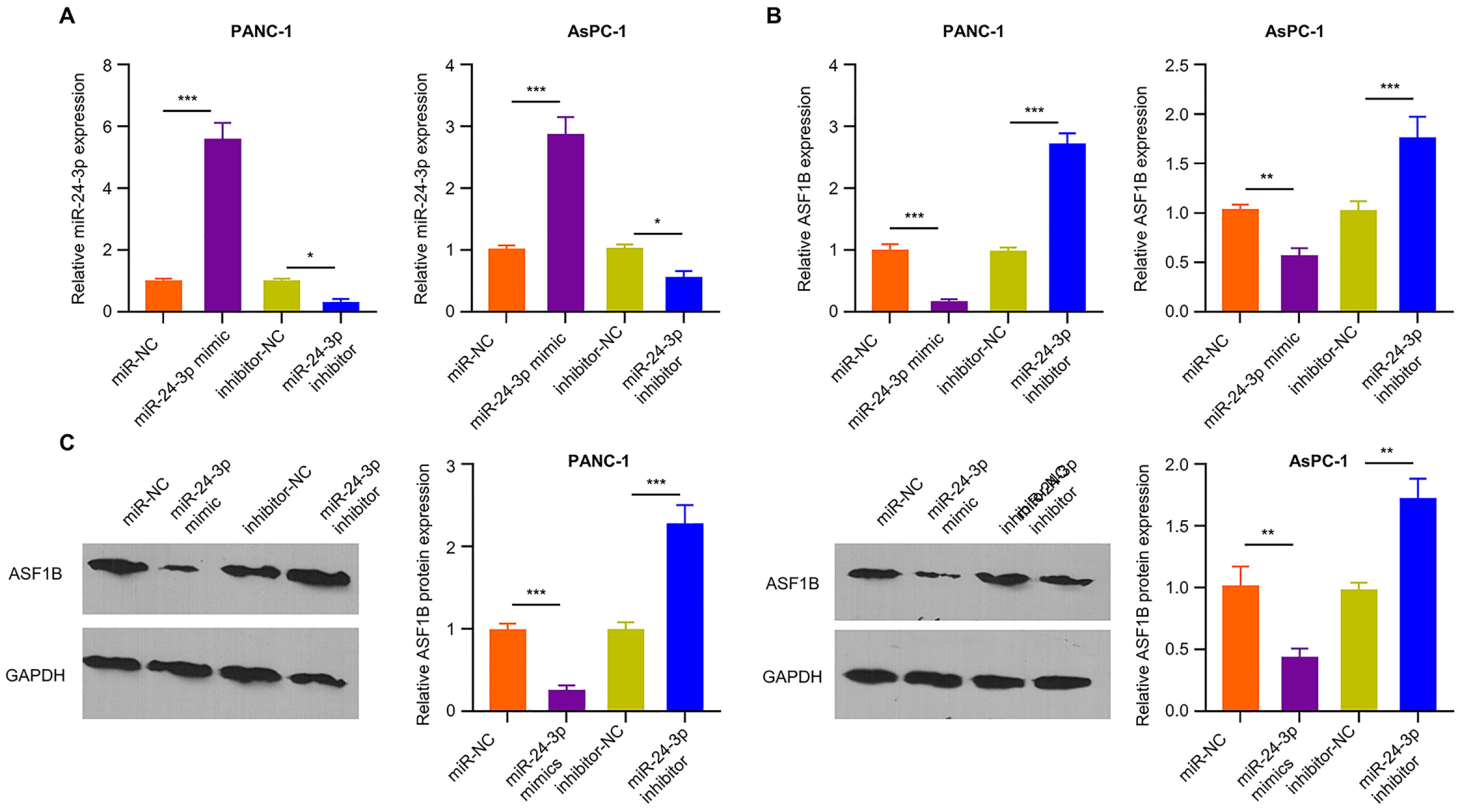
Overexpression of miR-24-3p suppresses ASF1B level in PDAC cell lines. **A** miR-24-3p level was measured after the transfection of miR-24-3p mimic or inhibitor. **B**, **C** ASF1B levels in AsPC-1 and PANC-1 cells transfected with miR-24-3p mimic or inhibitor were examined by qRT-PCR and WB. The data are summarized as mean ± SD. *p < 0.05, **p < 0.01, ***p < 0.001

To confirm the regulatory role of miR-24-3p in ASF1B expression, miR-24-3p mimic or inhibitor was transfected into AsPC-1 and PANC-1 cells to overexpress or reduce miR-24-3p expression (Fig. 4A). miR-24-3p mimic transfection remarkably decreased ASF1B expression at both mRNA and protein levels (Fig. 4 B and C), while miR-24-3p inhibitor transfection significantly increased ASF1B protein and mRNA levels (Fig. 4B and C). The above data indicate that miR-24-3p negatively regulates miR-24-3p expression.

### miR-24-3p Overexpression Attenuates the Malignancy of PDAC Cells, Which is Partially Rescued by ASF1B Overexpression

**Fig. 5:**
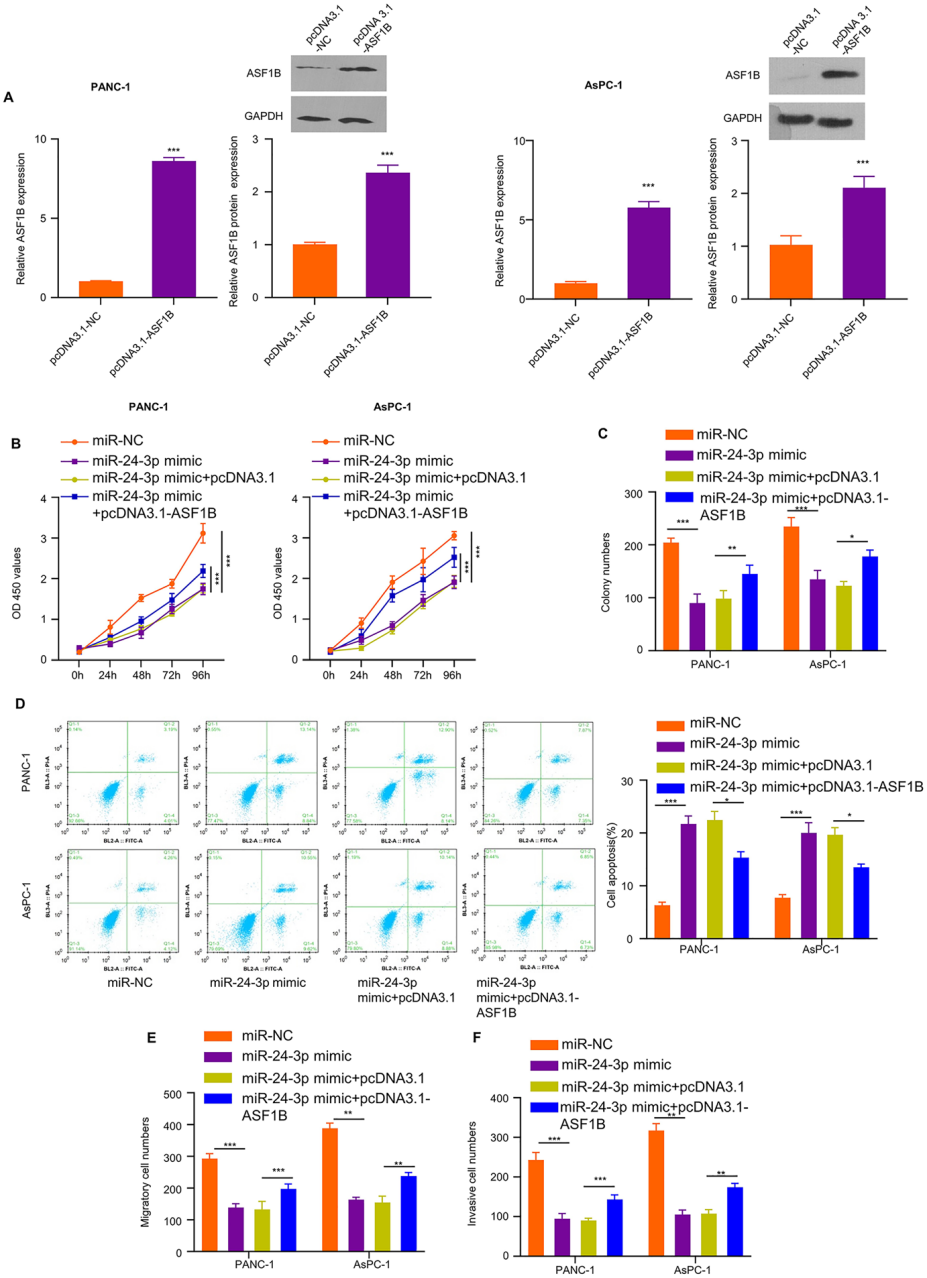
ASF1B overexpression attenuates the effects of miR-24-3p overexpression. **A** ASF1B mRNA and protein levels in AsPC-1 and PANC-1 cells after the transfection of ASF1B expression plasmid (pcDNA3.1-ASF1B) or empty vector (pcDNA3.1-NC) were analyzed by qRT-PCR and WB. AsPC-1 and PANC-1 cells were transfected with miR-NC, miR-24-3p mimic, miR-24-3p mimic + pcDNA3.1 vector or miR-24-3p + pcDNA3.1-ASF1B, and function assays including **B** CCK-8 **B** cell proliferation, **C** colony formation assay, **D** apoptosis detection, **E** migration and **F** invasion assays were performed in the above cells. The results are summarized as mean ± SD. *p < 0.05, **p < 0.01, ***p < 0.001

We next attempted to investigate the role of miR-24-3p in the regulation of ASF1B-dependent malignant phenotype in PDAC cells. pcDNA3.1-ASF1B expression vector or empty vector was transfected into AsPC-1 and PANC-1 cells, and the upregulation of ASF1B was confirmed through qRT-PCR and WB (Fig. 5A). Then AsPC-1 and PANC-1 cells were transfected with miR-NC, miR-24-3p mimic, miR-24-3p mimic + pcDNA3.1 vector or miR-24-3p + pcDNA3.1-ASF1B, and function assays including CCK-8 cell proliferation, apoptosis detection, colony formation assay, invasion and migration assays were performed. miR-24-3p mimic transfection significantly impaired cell proliferation, while ASF1B overexpression partially rescued cell proliferation (Fig. 5B, C). In contrast, miR-24-3p mimic transfection significantly increased the events of apoptosis; while ASF1B overexpression reduced apoptosis in PDAC cells (Fig. 5D). In addition, miR-24-3p mimic transfection also suppressed cell migration and invasion, while the co-transfection of ASF1B expression plasmid partially restored migratory and invasive abilities (Fig. 5E, F). These data indicate that miR-24-3p overexpression undermines the malignancy of PDAC cells, which is partially rescued by ASF1B overexpression.

### ASF1B Silencing Impairs the EMT Process in PDAC Cells

**Fig. 6:**
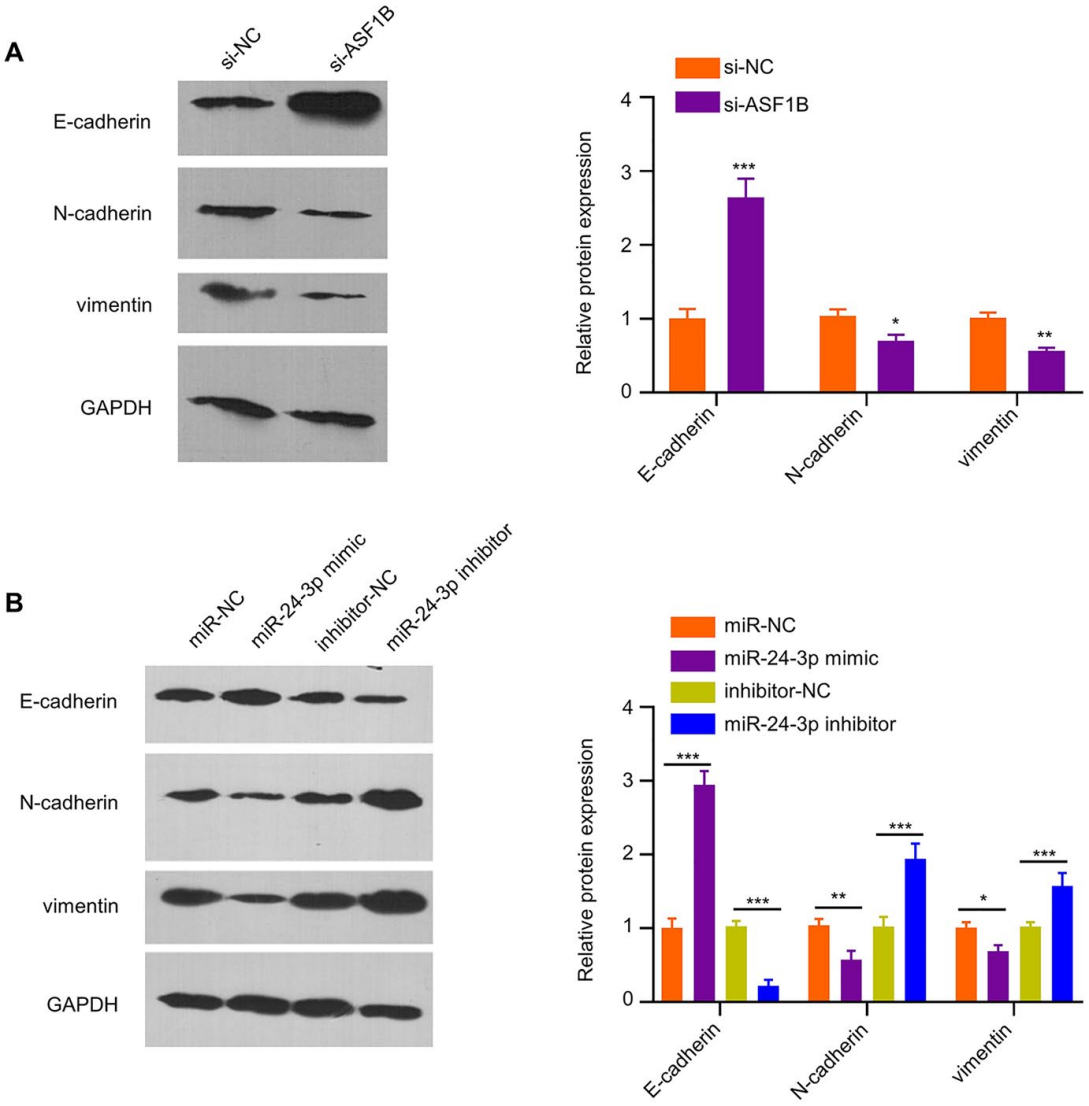
EMT is attenuated by the silencing of ASF1B. **A** EMT-related markers (E-cadherin, vimentin, N-cadherin) in AsPC-1 cells were examined by WB after ASF1B silencing in AsPC-1 cells. **B** EMT-related markers (E-cadherin, vimentin, N-cadherin) in AsPC-1 cells transfected with miR-24-3p mimic or inhibitor was measured by WB. The results represent mean ± SD. *p < 0.05, **p < 0.01, ***p < 0.001

EMT plays a critical role in the metastasis and progression in pancreatic cancer (Hamada et al. 2012). To determine whether ASF1B regulates EMT process in PDAC cells, we carried out WB to analyze EMT-related markers (E-cadherin, Vimentin, N-cadherin) in AsPC-1 cells upon ASF1B silencing. Silencing ASF1B in AsPC-1 increased E-cadherin level, but redcued vimentin and N-cadherin levels (Fig. 6A), which indicates ASF1B is required for the EMT status in PDAC cells. Next, AsPC-1 cells were transfected with miR-24-3p inhibitor or mimic to reduce or overexpress miR-24-3p level. It was found that E-cadherin level was upregulated and vimentin and N-cadherin levels were downregulated after the transfection of miR-24-3p mimic, and miR-24-3p inhibitor transfection showed the opposite effects (Fig. 6B). These results suggest that miR-24-3p/ASF1B axis regulates EMT process in PDAC cells.

## Discussion

Pancreatic cancer is a very aggressive malignancy with rapid progression and poor prognosis (Goral 2015), and it is usually diagnosed at advanced or metastatic stages (Chiorean and Coveler 2015). Understanding the molecular the mechanisms underlying its progression, invasion and metastasis can provide insights into the development of novel interventions. In this study, we demonstrated that ASF1B is highly expressed in pancreatic cancer and its high expression promotes the migration, invasion and EMT in PDAC cells. Consistently, Huang et al. (2022) applied WGCNA analysis using pancreatic cancer datasets in TCGA and found that the upregulation of ASF1B is correlated with the occurrence of pancreatic cancer. In addition, high expression of ASF1B level was also found to be associated with poor prognosis in different cancers (Hu et al. 2021; Feng et al. 2021). Particularly in hepatocellular carcinoma and male thyroid cancer, ASF1B was proposed as a potential target and immunological marker for tumor therapy (Ouyang et al. 2021; Zhang et al. 2022; Qiu et al. 2022). Meanwhile, the high expression of ASF1B was closely related to advanced TNM stage, larger tumor size and more lymph node metastases in PDAC patients, suggesting that ASF1B upregulation contributes to the progression of PDAC.

Our functional experiments further showed that ASF1B functions as an oncogene in PDAC cells, since the upregulation of ASF1B could promotes the malignancy of PDAC cells while silencing ASF1B undermines the malignancy. Similarly, the oncogenic role of ASF1B has been reported in multiple cancers, such as breast cancer, clear renal cell carcinoma and prostate cancer (Corpet et al. 2011; Han et al. 2018; Jiangqiao et al. 2019). In a pancreatic cancer study, ASF1B knockdown can also suppress the tumor progression and improved the efficacy of cisplatin in the treatment of pancreatic cancer (Kim et al. 2022). Therefore, ASF1B may serve as a general oncogene to promote the malignancy phenotypes in different cancers.

EMT is the process of cellular transformation from the epithelial phenotype into the mesenchymal phenotype, which is involved in tumor invasion and metastasis (Hugo et al. 2007; Li et al. 2019). During EMT, cells lose epithelial cell polarity and E-cadherin expression level and cell junctions decrease, which is accompanied by an enhanced migration and invasion ability (Craene and Berx 2013). The early metastasis of pancreatic cancer is closely related to the EMT process (Beuran et al. 2015). ASF1B upregulation is previously suggested to be correlated with the EMT in lung cancer (Wu and Jie 2021). In our work, we showed that silencing ASF1B reduced the expression of mesenchymal markers (vimentin and N-cadherin), but increased the expression of E-cadherin. These data suggest that ASF1B is a positive regulator of EMT process in PDAC.

EMT process can be regulated by multiple miRNAs (Pan et al. 2021). miRNAs usually recognize the 3'UTR of target mRNAs and suppress the protein translation or induce the degradation of target mRNAs (Cannell et al. 2008). In our work, miR-24-3p was identified as a negative regulator of ASF1B. The miR-24-3p-ASF1B axis not only regulates the malignant phenotype of PDAC cells, but also modulates the EMT process. These data suggest that miR-24-3p regulates EMT process and the malignant phenotype of pancreatic adenocarcinoma by regulating ASF1B expression. miR-24-3p showed aberrant expression and has been proposed as a prognostic indicator in different tumors (Wang et al. 2020). miR-24-3p can act as a tumor-suppressor in multiple cancers, including the inhibition of EMT and apoptosis induction in tumor cells (Jing et al. 2020). In pancreatic cancer, miR-24-3p was reported to suppress the progression of PDAC by targeting LAMB3 (Huang et al. 2019). Together, our data and previous studies altogether support the tumor-suppressing role of miR-24-3p in pancreatic cancers. However, the role of miR-24-3p/ASF1B in the progression of PDAC needs to be validated in animal models.

In summary, ASF1B shows high expression in PDAC tissues and cells, which promotes EMT process and the malignant phenotype of PDAC cells. miR-24-3p serves as an upstream negative regulator of ASF1B, which is downregulated in PDAC tissues and cells. Our data suggest that targeting ASF1B/miR-24-3p axis could serves a plausible therapeutic strategies in the management of PDAC.

## Data Availability

The datasets used and/or analyzed during the current study are available from the corresponding author via e-mail request.

## Abbreviations

ASF1B: Anti-silencing function protein 1 homolog B
PDAC: Pancreatic ductal adenocarcinoma
EMT: Epithelial−mesenchymal transition
qPCR: Real-time quantitative PCR
OS: Overall survival
DFS: Disease-free survival
ASF1: Anti-silencing function 1
IRS: Immunoreactive score
SI: Staining intensity
PP: Percentage of positively
PVDF: Polyvinylidene difluoride
3’UTR: 3' Noncoding region
